# Fracture resistance of endodontically treated teeth, restored with two post-core systems in different post space diameters (in vitro study)

**DOI:** 10.1186/s12903-023-03730-4

**Published:** 2023-12-11

**Authors:** Kholoud B. Saad, Samir I. Bakry, Rewaa G. AboElhassan

**Affiliations:** 1 Division of Fixed Prosthodontics, Department of Conservative Dentistry, Faculty of Dentistry, Alexandria University, Alexandria, Egypt; 2 Department of Conservative Dentistry, Faculty of Dentistry, Alexandria University, Alexandria, Egypt; 3 Department of Conservative Dentistry, Faculty of Dentistry, Alexandria University, Alexandria, Egypt

**Keywords:** Zirconia, Glass-fiber, Post, Fracture resistance, Diameter

## Abstract

**Background:**

Fracture resistance of post-core restoration depends on the design of the post, post diameter, post length, the type of adhesive cement used along with material of the core. Despite the different studies concerning the effect of post space diameter on the fracture resistance of endodontically treated teeth, more information regarding fracture resistance and the effect of different post space systems/materials and diameters is required.

**Aim of the study:**

This study aimed to evaluate fracture resistance of endodontically treated teeth, restored with two post-core systems in different post space diameters.

**Materials and methods:**

Twenty freshly extracted maxillary central incisors were collected for this study. They were randomly divided into four groups according to intracanal post and its diameter. Group GN (glass-fiber post of 10-mm length and 1.3-mm diameter with composite core), group GW (glass-fiber post of 10-mm length and 1.75-mm diameter with composite core), group ZN (custom-made zirconia one-piece post-core of 10-mm length and 1.3-mm diameter) and group ZW(custom-made zirconia one-piece post-core of 10-mm length and 1.75-mm diameter).

Fracture resistance for all samples was evaluated using the universal testing machine under a static load. The data was collected and statistically analyzed using One-Way ANOVA test. Modes of failure were assessed using stereomicroscope for each group.

**Results:**

The highest mean fracture resistance was recorded in group GW (638.7 ± 285.1 N), followed by group ZW (598.5 ± 127.6 N), then GN group (442.8 ± 65.38 N). The lowest mean fracture resistance was recorded in group ZN (435.3 ± 117.3 N). One-Way ANOVA test revealed that there was no statistically significant difference in fracture resistance values among the groups.

**Conclusion:**

Post space diameter had an impact over the fracture resistance of endodontically treated teeth. Modulus of elasticity of post material had a major effect on the fracture resistance and mode of failure along with the restorability of the restored tooth. However, there was no statistically significant difference among the tested groups.

## Background

The restoration of endodontically treated teeth is controversial and remains a challenge to the dental clinicians, as a result of major loss of natural tooth structure due to dental caries or trauma; which is mandatory for the retention of the coronal restoration. The remaining tooth structure is the most crucial factor for the good prognosis as they are devoid of mechanical properties including loss of strength, fragility as well as the liability to fracture [1, 2].

Endodontically treated teeth are traditionally restored with a post-core and crown foundation system which depend mainly on the design, material and modulus of elasticity of dental posts as they play a major role in retention and fracture resistance of coronal restoration of endodontically treated teeth [3].

Posts are classified according to their material into metallic or tooth-colored posts. Tooth-colored posts are further classified into pre-fabricated and custom-made posts. Among the prefabricated type, fiber posts are the most appropriate to select as they acquire simple technique of application in addition to the optimum mechanical properties; dentin-like modulus of elasticity that aids in dissipation of occlusal forces and the decrease of incidence of root fracture [4, 5].

Fiber posts contain either carbon fibers or quartz fibers, embedded in epoxy or methacrylate resin matrix. The fibers are parallel to the long axis of the post with diameter ranging between 6 to 15 mm. While the number of fibers range between 25 and 35 per mm^2^ in respect to post type and cross-sectional surface. So as a result, 30–50% of the area is filled with fibers when a transverse section is seen [4, 6, 7]. Nevertheless, leakage and contamination of the root canal might occur due to the high flexibility of posts [4, 8].

Custom-made tooth-colored posts include ceramic and zirconia posts. They are used when high esthetic demand is required in the anterior zone of teeth to prevent long term discoloration. However, these posts show difficult retrievability in the retreatment cases as the removal require rotary instruments which might lead to root fracture or perforation [4, 8, 9].

Zirconia post and core system offers chemical stability and its similarity to natural tooth structure whereby the tooth-colored translucency of all-ceramic crowns that provides high stiffness and distributes stresses better to the root; providing greater clinical longevity, light transmission and radio-opacity as well as the promising esthetic restorative outcome to the patients [10–13]. The alteration or any minute modification in post space preparation either in width or length might alter the properties of glass-fiber post and zirconia post and core system which might lead to changing the fracture resistance of post or endodontically treated teeth themselves [3].

Grewal et al. and Khaldi et al. stated that the success of various post systems depend on length, diameter, design of the post, canal shape and preparation, ferrule, luting cement, technique of cementation along with its location in the dental arch and proper retention of the post is mandatory in order to sustain the vertical occlusal forces [4, 14].

Sughaireen et al. and Mou et al. recommended the optimum diameter of the post preparation to stay at one-third of root’s diameter or have 1:4 ratio approximately. This wide preparation of posts aided in resisting occlusal forces but led to root fracture [15, 16]. On the other hand, Kaur et al., Nokar et al., Alomari et al. and Huysmans et al. stated that the larger the diameter of the post, the increased risk of root fracture, so a specific diameter is mandatory to provide optimal physical properties and prevent root fracture or failure of the post when being subjected to functional and parafunctional forces [17–21].

In spite of different statements concerning the effect of post space diameter on the fracture resistance of endodontically treated teeth, more information regarding fracture resistance and the effect of different post space systems/materials and diameters is required. This study aimed to evaluate fracture resistance of endodontically treated teeth, restored with two post-core systems in different post space diameters. The null hypothesis of this study was that there would be no significant difference between fracture resistance of endodontically treated maxillary central incisors, restored with glass-fiber posts and composite resin core and zirconia post-core one-piece restoration in different post space diameters.

## Materials and methods

This study was in-vitro, parallel controlled in which fracture resistance and failure mode of four parallel groups were examined. It was held at the Conservative Dentistry Department laboratory at the Faculty of Dentistry, Alexandria University, Egypt. A sample size was calculated using a sample size calculation program(G*Power version 3.1.9.2, Department of Biomedical Informatics and Medical Statistics, Medical Research Institute, University of Alexandria, Egypt.) [22] where α = 0.05 and power of 80% and the mean and standard deviation were taken from a previous study [23]. At least 5 samples were assigned for each group; this minimal sample size calculation was done depending on a previous study [23], the number of groups in this research is 4, so the total sample size is 20 specimens.

For this study, 20 freshly extracted, for orthodontic or diabetic purpose, maxillary central incisors were collected and preserved in normal sterile saline until the onset of use. For teeth standardization, teeth selected for this study had nearly same size and shape with intact coronal and apical structure as well as root canal diameters that aided in drilling of post spaces. Periapical radiographs were taken for each specimen to verify the straight pathway of the root canals. Each specimen was measured using a Digital Caliper (Sedradent, TDV Co. Pomerode, Santa Catarina, Brazil) to insure size range at the cervical line area of 6 ± 1 mm bucco-lingually and 5 ± 1 mm mesio-distally and root length from cemento-enamel junction to the root apex of 13 ± 0.5 mm. The specimens with similar root length and diameters were selected and stored in sterile saline solution until use.

Teeth with extensive root caries, fracture, and internal/external cracks/resorption were excluded from this study. Also, teeth with previous endodontic treatment or severe apical curvature were excluded.

### Teeth preparation

The selected teeth root lengths were measured from the cervical line of teeth to the apex using Digital caliper (Sedradent, TDV Co. Pomerode, Santa Catarina, Brazil); they were more or less 13 mm long (± 0.5 mm). Teeth were then mechanically cleaned with aid of hand scaler (Nordent Manufacturing Inc., 610 Bonnie Lane, Elk Grove Village, IL 60007, T: 800.966.7336 US & Canada)to remove remaining dental plaque, calculus, and periodontal tissues [23, 24].

Eighteeth curing pen (Changzhou Sifary Medical Technology Co., Ltd.) was used to transilluminate the specimens at light intensity of 600 mW/cm2 and wavelength of 385 nm–410 nm to ensure they were devoid of any internal or external crack lines [23, 25]. After that, crowns were sectioned 2-mm above the cemento-enamel junction with a diamond disc (Komet Dental. Gebr. Brasseler GmbH & Co. KG Trophagener Weg 25.32657 Lemgo. Germany) mounted on high-speed handpiece under continuous coolant to standardize the remaining root length at ±13 mm. A ferrule of 2-mm height and 1.5-mm depth was prepared in all specimens [26].

**Fig. 1:**
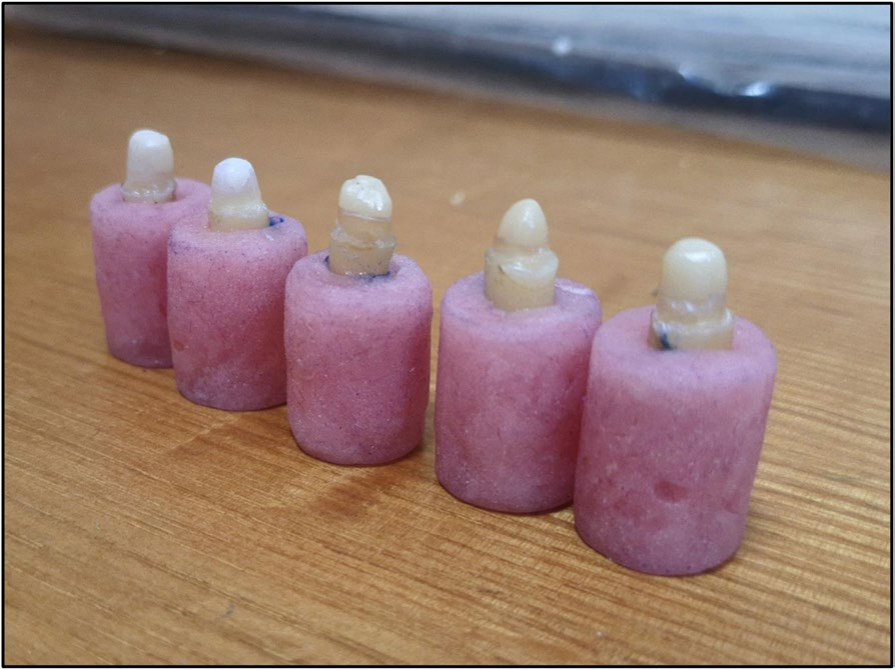
Specimens held in acrylic resin blocks

Teeth were embedded in auto-polymerizing acrylic resin blocks, in cylindrical metallic molds, along with their vertical long axis; leaving 2 mm coronal structure out of each block. Teeth were then removed, light-body ZHERMACK Elite HD+(Zhermack S.p.A., Via Bovazecchino, 100 | 45,021 Badia Polesine (RO) ITALY) was injected all around the roots of specimens and inside their corresponding acrylic blocks and each specimen was repositioned into its specific block. Excess material was removed before complete setting. That step allowed the simulation of the periodontal ligament space in a thickness of 0.5 mm [23, 24] Fig. 1.

### Access opening and root canal preparation of specimens

Preoperative periapical radiograph was taken for each specimen to ensure absence of any kind of root canal calcifications, internal resorption or presence of pulp stones or accessory canals. Rose-head bur and Endo-Z(Komet Dental. Gebr. Brasseler GmbH & Co. KG Trophagener Weg 25.32657 Lemgo. Germany) were mounted on high-speed handpiece, respectively, for access cavity preparation. Root canal length determination was done by inserting number 10 k-file (MANI, INC., Tochigi-Ken, Japan^.^) into the root canal, until reaching the root apex. Each length was verified through periapical x-ray for each specimen [23, 26].

WaveOne reciprocating single file(Maillefer-Dentsply, Ballaigues, Switzerland.) was used for mechanical preparation of root canals up to the full working length. The tip size was ISO 40 with an apical taper of 8% that reduced towards the coronal end. Regarding irrigation, 2.5% Sodium Hypochlorite irrigation solution using 30-gauge side-vented irrigation needle(SUNGO Certification Company Limited, RM101, Maple House, 118 High Street Purley, London, England.); after every instrument change to ensure the complete elimination of any dentinal debris which might cause blockage of the canals [23–26].

Root canals were dried using #40 paper points until complete drying of the canals was achieved. Cold lateral condensation technique was followed to obturate all the canals using ISO #40 gutta-percha(Metabiomed Co. LTD, South Korea.) cone, ISO #30 gutta percha(Metabiomed Co. LTD, South Korea.) accessory cones and ADseal sealer(Metabiomed Co. LTD, South Korea.) to the full working length. Access cavities were then filled with Tetric N-ceram nanohybrid composite(Ivoclar Vivadent Dental Products, Liechtenstein, Germany.) temporarily to be stored in laboratory incubator at 37 °C and 100% relative humidity for at least 2 days; allowing the complete setting of endodontic sealer [23, 24, 26].

### Grouping of specimens

The total number of specimens was randomly divided into two main groups (*N* = 10), according to the type of post/core material used.

**Group Z**: 10 teeth restored with CAD/CAM zirconia post-core as one-piece unit. **Group G**: 10 teeth restored with Glass-fiber post and composite resin core. Each group was further subdivided into two subgroups (*N* = 5) according to the diameter of post space preparation. **Subgroup GN:** Five teeth restored with long glass-fiber post with narrow diameter of 1.3 mm, and composite resin core. **Subgroup GW:** Five teeth restored with long glass-fiber post with wide diameter of 1.75 mm, and composite resin core. **Subgroup ZN:** Five teeth restored with long CAD/CAM zirconia post with narrow diameter of 1.3 mm, and zirconia core as one-piece unit. **Subgroup ZW:** Five teeth restored with long CAD/CAM zirconia post with wide diameter of 1.75 mm, and zirconia core as one-piece unit.

### Post space preparation

After the random division of specimens, the access cavities were reopened again with round bur mounted on a high-speed handpiece until completely removed. Post space preparation protocol was followed including removal of gutta percha from the two coronal thirds of the roots; which was approximately 10 mm long, leaving ±4 mm of gutta percha apically as an apical seal, indicating a long post space using peeso reamer drills (MANI, INC., Tochigi-Ken, Japan) that were mounted on a low-speed handpiece, length adjusted by endodontic ruler and then used in the order of #1, #2, #3 and #4 sequentially, to achieve a standardized post space preparation; 0.7, 0.9, 1.1 and 1.3 mm in diameter respectively. 10 mm of gutta percha was removed from all canals leaving 4 mm of coronal seal, to standardize the length of preparation in all specimens [23, 24, 26].

#### For the narrow glass-fiber post space preparation (subgroup GN)

Using no. 1 shaping drill of HAHNENKRATT-Contec glass-fiber post kit(E.HAHNENKRATT GMBH | Benzstr. 19 | DE-75203 Königsbach-Stein, Germany), the white calibration drill of 1.3 mm diameter was used to prepare a narrow post space, in respect of adjusting the drill’s length to 10 mm [23, 24, 26].

#### For the wide glass-fiber post space preparation (subgroup GW)

Using no. 3 shaping drill of HAHNENKRATT-Contec glass-fiber post kit, the blue calibration drill of 1.75 mm diameter was used to prepare a wide post space, in respect of adjusting the drill’s length to 10 mm [23, 24, 26].

Regarding the composite resin core that was built in both subgroups GN and GW, it had dimensions of 4-mm labio-palatally, 4-mm mesio-distally and 4-mm inciso-cervically. These composite cores were then built after the cementation procedure of glass fiber posts [23].

#### For the narrow zirconia post space preparation (subgroup ZN)

For a standardized post space preparation and to ensure the similar and precise dimensions of both glass-fiber post and custom-made zirconia post, the same calibration drill of group GN was also used in ZN subgroup.

No. 1 white calibration drill of 1.3 mm diameter was used to prepare a narrow post space, in respect of adjusting the drill’s length to 10 mm [23, 24, 26].

#### For the wide zirconia post space preparation (subgroup ZW)

Using the shaping drills of Contec-HAHNENKRATT glass-fiber post kit, no. 3 blue calibration drill of 1.75 mm diameter was used to prepare a wide post space for the ZW subgroup, in respect of adjusting the drill’s length to 10 mm [23, 24, 26].

Periapical radiograph was taken for each specimen to insure complete removal of root canal filling material as well as the fitting of the corresponding glass-fiber post in both subgroups ZN and ZW [23, 24, 26].

### Direct post-core pattern fabrication (subgroups ZN and ZW)

Post spaces were irrigated with 2.5% NaoCl, normal saline and 70% alcohol then partially dried with air/water syringe then completely dried using endodontic paper points. Plastic posts were firstly inserted into specimens’ canals to check its fitting passively, each plastic post was marked to the predetermined length; 10 mm length, then was inserted back to confirm the proper seating [13, 23, 27, 28].

Light-cured universal modelling acrylic resin of gel-consistency (Sedradent, TDV Co. Pomerode, Santa Catarina, Brazil)was injected into each prepared canal around the plastic post, slight bumping motion was done without any excessive pressure, to the predetermined length. Light-curing of the material was done for 20 seconds and 1500 mW/cm^2^. The pattern was pulled out carefully to ensure the continuous smooth surface, being completely coated with the resin material that was devoid of any air-bubble entrapment or distortion, to ensure dimensional standardization in the four subgroups, either for narrow or wide subgroups. Finally, it was tested again for proper fitting [13, 27–30].

The core was built with the same material; light-cured universal modelling acrylic resin of paste-consistency (Sedradent, TDV Co. Pomerode, Santa Catarina, Brazil) with the same dimensions of core of GN and GW subgroups. After obtaining the proper dimensions, the core was finished and polished using composite finishing and polishing burs [13, 27–30].

### Scanning of resin pattern

The fabricated direct resin patterns were held upside down, the highest point of the core curvature was placed over the stiff clay that was supported in metallic circular molds to ease the scanning procedures without deformation of the pattern and gaining the correct dimensions throughout the scanning process.

### Designing and milling of zirconia one-piece post-core restoration

ZirkonZahn blank(Prettau 2 Dispersive, Zirkonzahn Srl, Via An Der Ahr 7–39,030 Gais(Bz),Val Pusteria-South Tyrol–Italy)was used for the milling process to fabricate the zirconia post-core samples into the required dimensions, leaving 2-mm ferrule all around which permitted zirconia core to mimic same dimensions of composite cores in both groups GN and GW, by using ED5X(EMAR MILLS,C2, Industrial Complex, 10th of Ramadan City Asharqia, Egypt) milling machine, with prime water cooling system to produce one-piece post-core unit with respect to different diameters of tested groups (LN and LW). Nevertheless in this study, dry milling was performed in conjugation to dust extraction unit to vacuum any dust or particles resulting from the milling process. The milling process took about 10 minutes for each specimen, according to manufacturer’s instructions.

### Sintering of the zirconia post-core restorations

MV-R (MIHM-VOGT GmbH & Co.; Friedrich-List-Str. 876,297 Stutensee-Blankenloch) sintering furnace was used to sinter zirconia samples that took about 8 hours, as it required 4 hours to raise from room temperature up to 1550 ^o^c, then it was kept for 2 hours in 1550 ^o^c temperature, then it took 2 hours to return back from 1550 ^o^c to room temperature gradually in order to cool down completely, following the manufacturer’s instructions. Zirconia restorations were checked for proper fitting into their corresponding samples.

### Cementation of glass-fiber posts in subgroups GN and GW

Post spaces were irrigated with 2.5% NaoCl, normal saline and 70% alcohol, dried and etched with 37% phosphoric acid(Metabiomed Co. LTD, South Korea) then rinsed with water then completely dried by endodontic absorbing paper points(Metabiomed Co. LTD, South Korea) [23, 26, 31–34].

Calibra Universal Self-Adhesive resin cement (Maillefer-Dentsply, Ballaigues, Switzerland) was used for cementation of each specimen with changing injection tip, with respect to different diameters for each subgroup. Following the manufacturer’s instructions, the auto-mixing tip was attached then injection of the cement was done into the post spaces as well as all around posts’ surfaces. It was light-cured for 20 seconds. The excess cement was removed using composite finishing burs(Komet Dental. Gebr. Brasseler GmbH & Co. KG Trophagener Weg 25.32657 Lemgo. Germany) mounted on high-speed handpiece.

### Composite resin core for subgroups GN and GW

After cementation of the glass-fiber posts, Tetric N-ceram nano-hybrid composite (Ivoclar Vivadent Dental Products, Liechtenstein, Germany) was used to build the core for both subgroups GW and GN. It was dome-shaped with dimension of 4-mm circumferentially [23, 31].

### Luting the fabricated zirconia post-core for subgroups ZN and ZW

Calibra Universal Self-Adhesive resin cement was also used to cement the zirconia post-core restorations into their corresponding post spaces in each group, in respect to different diameters for each subgroup.

### Tests

#### Fracture resistance test

**Fig. 2:**
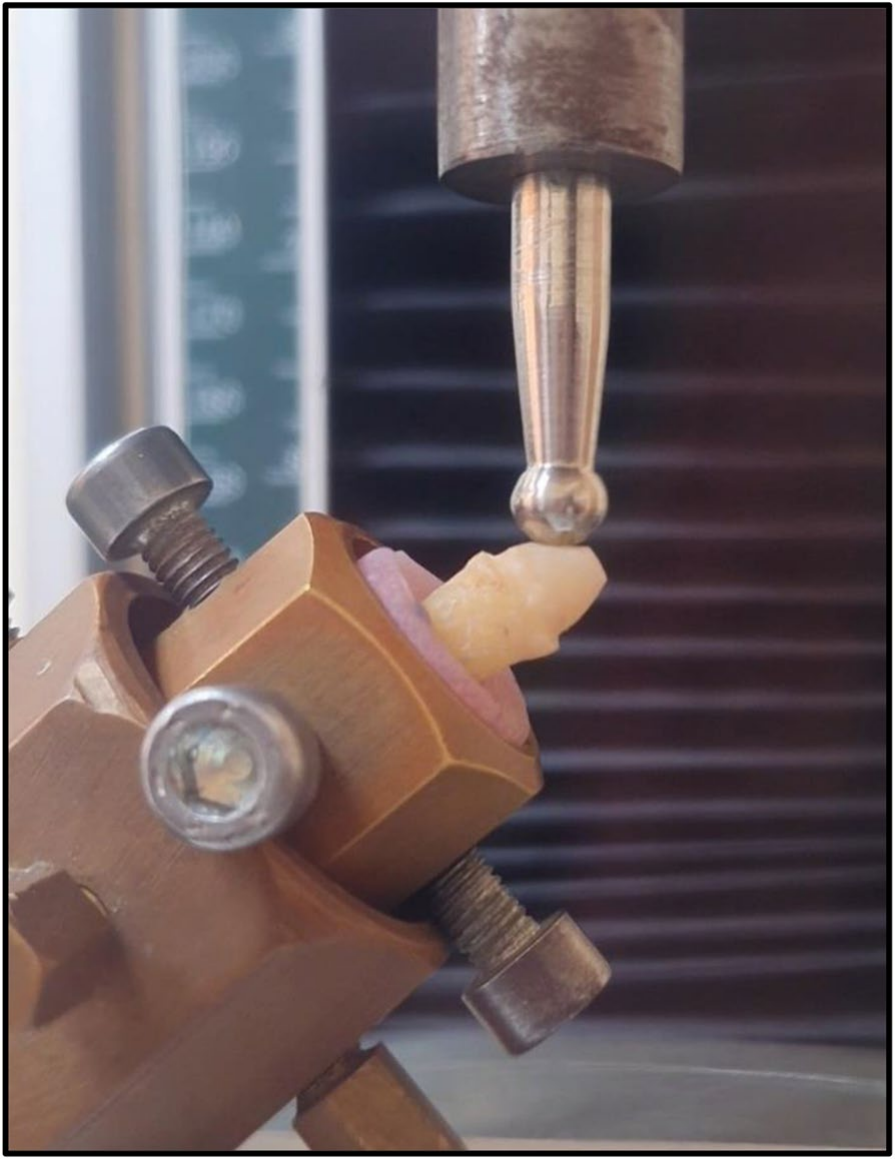
Specimens with acrylic resin blocks held in metallic molds to be tested by universal testing machine

Samples with the acrylic resin blocks were held in metallic molds for testing. Specimens were loaded at 3 mm below the incisal part of the core palatally at an angle of 45^o^ to the long axis of the teeth. Then each specimen was loaded to test its fracture resistance in the Tinius Olsen 5ST (6 Perrywood Business Park, Honeycrock Lane, Salfords (Near Redhill), Surrey RH1 5DZ, England) universal testing machine with a crosshead that had 6-mm diameter and speed of 1 mm/min until catastrophic failure happened. The testing machine automatically recorded the fracture force in Newton by Tinius Olsen Horizon software, version 10.2.4.22 [35, 36] Fig. 2.

#### Assessment of mode of failure

To determine the type of failure, the failed teeth were examined under a stereomicroscope (SZ1145TR Olympus; Japan 1990) by using software (Toup view, version 3.7). It was classified as follows [35, 36]:

- Favorable or restorable failure: When the failure or fractured line or area was found to be above the cervical line of restored teeth.
- Non-favorable or non-restorable or catastrophic failures: were present when the fracture line or failure occurred at the cervical line or extended beyond it. Vertical root fracture is also included.

### Statistical analysis

Comparison between the four study groups was done using One-Way ANOVA test. Data were fed to the computer and analyzed using IBM SPSS software package version 20.0***.*** **(**Armonk, NY: IBM Corp**).** Quantitative data were described using range (minimum and maximum), mean, and standard deviation. Significance of the obtained results was judged at the 5% level. Significance was set at *P* value ≤0.05.

## Results

**Fig. 3:**
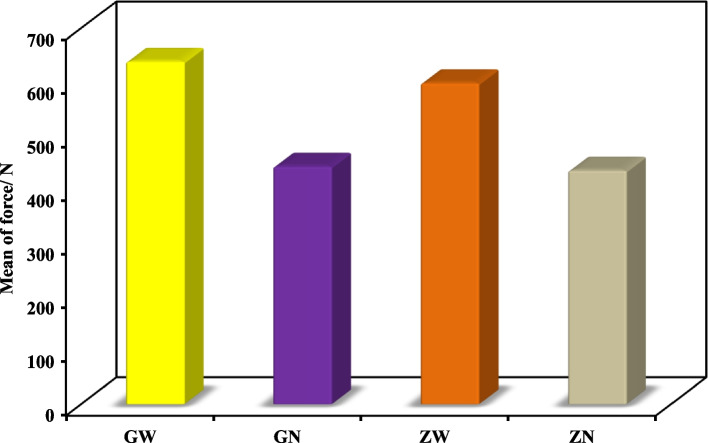
Comparison between the different studied groups according to Force/ N

**Fig. 4:**
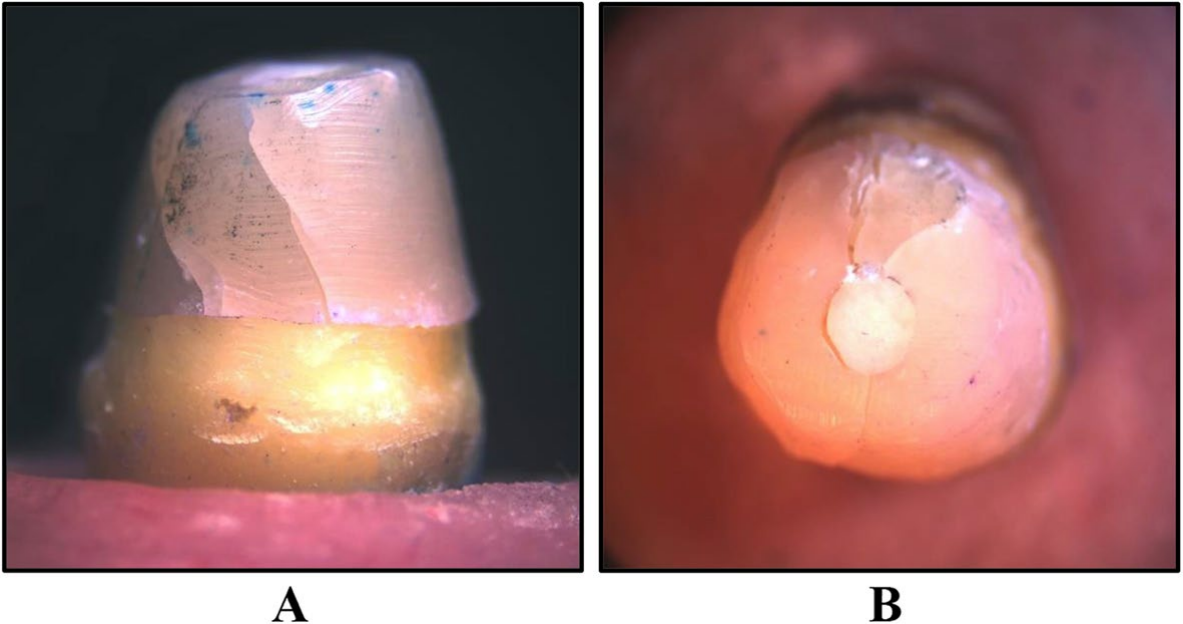
Stereomicroscopic evaluation at magnification of × 18 showing favorable failure of group G **A** Proximal view of a failed specimen showing crack propagation in the core only with integrity of cervical line. **B** Incisal view showing debonding between the core and post and presence of crack lines confined only to the core

**Fig. 5:**
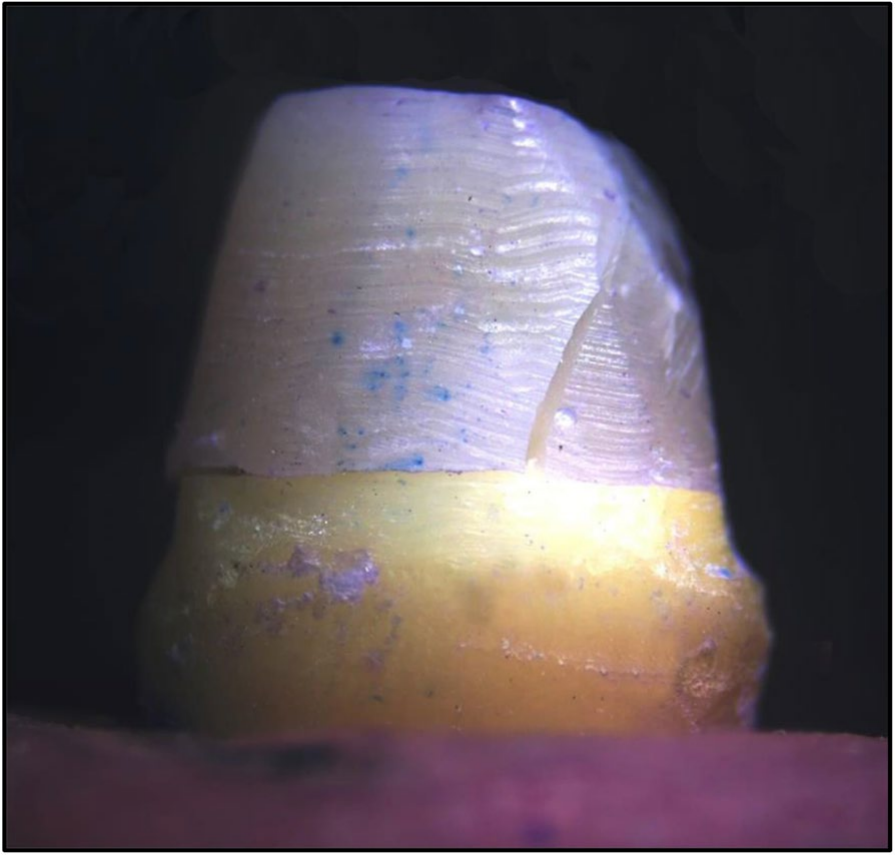
Another specimen showing cracking and defragmentation of the core with integrity of the post and cervical line of the tooth

The mean fracture resistance of all groups; GW, GN, ZW and ZN presented the following values:

- The highest mean fracture resistance was recorded in group GW (638.7 ± 285.1 N), followed by group ZW (598.5 ± 127.6 N), then GN group (442.8 ± 65.38 N). The lowest mean fracture resistance was recorded in group ZN (435.3 ± 117.3 N). One-Way ANOVA test revealed that there was no statistically significant difference in fracture resistance values among the groups. As shown in Table 1 and Fig. 3.
- The failure modes as a result of the static loading were evaluated with a stereomicroscope, under magnification of × 18 and × 20:
- Favorable/restorable fractures were exhibited in group G. Figs. 4 and 5
- Catastrophic/non-favorable fractures were observed most in group Z, and none in group G.

**Table 1:** Comparison between the different studied groups according to Force/ N

Force/ N	GW	GN	ZW	ZN	F	p
Min. – Max.	441.4–1081.3	354.3–520.4	497.7–766.0	295.4–569.4	1.909	0.169
Mean ± SD.	638.7 ± 285.1	442.8 ± 65.38	598.5 ± 127.6	435.3 ± 117.3		

*SD* Standard deviation, *F* F for One way ANOVA test

p: *p* value for comparing between the studied groups

As shown in Fig. 6.

**Fig. 6:**
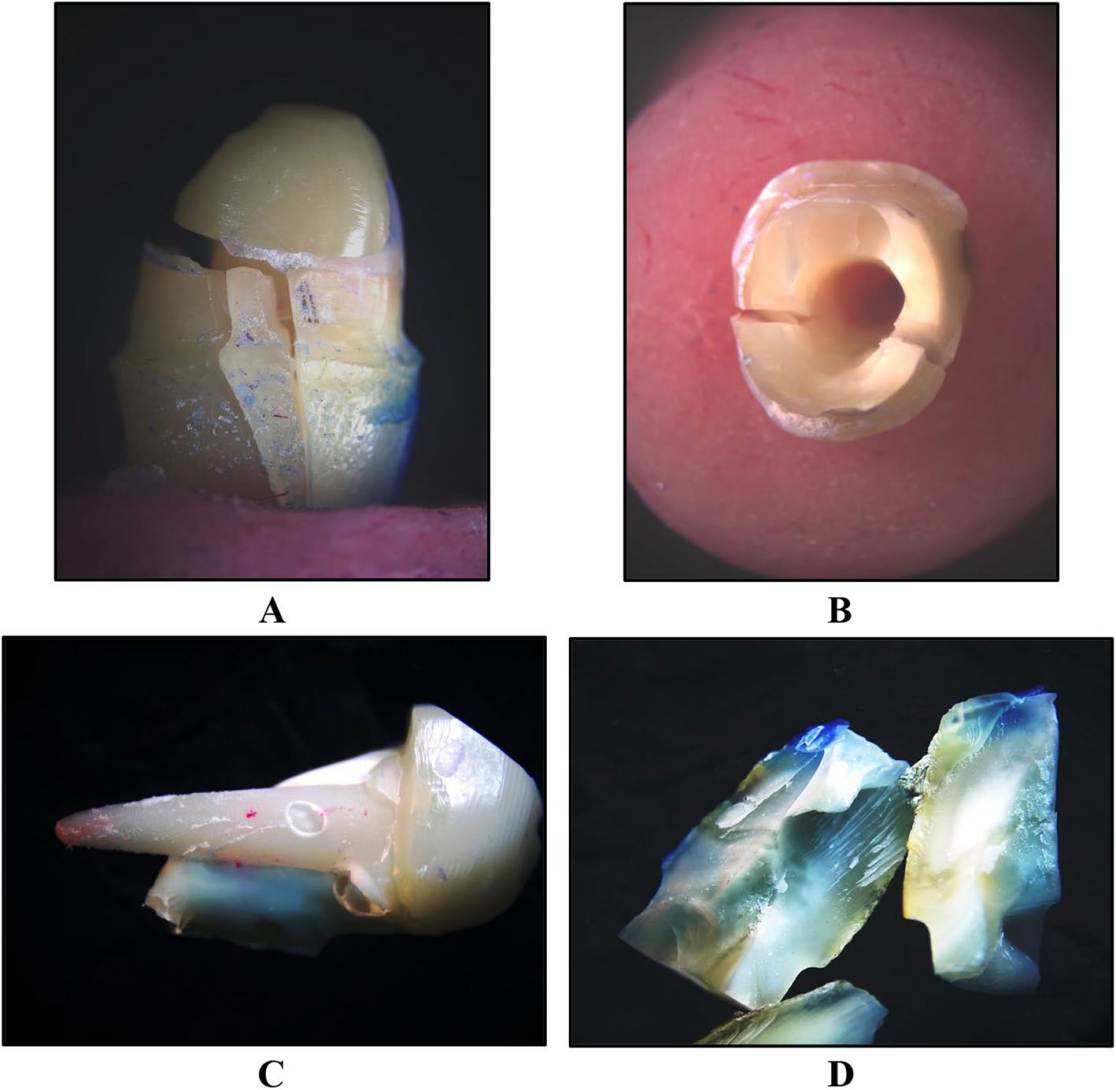
Stereomicroscopic evaluation at × 20 magnification showing catastrophic failure of group Z **A** Proximal view showing non- favorable fracture involving cervical line of the tooth with vertical root fracture. **B** Incisal view showing major cracks propagation. **C** Survival of the zirconia post-core with total loss of the specimen. **D** Remaining fragments resulting from catastrophic failures

## Discussion

When the remaining tooth structure of endodontically treated teeth cannot provide sufficient support and retention for restoration; remaining less than 50% of tooth structure, post and core is beneficial. So the rehabilitation of pulpless teeth is crucial in order to restore them esthetically, functionally and structurally for a successful restorative outcome and prognosis [37, 38].

Fracture resistance of post-core restoration depends on the design of the post, post diameter, post length, the type of adhesive cement used along with material of the post and core system material that aids in dissipation of occlusal forces along to the post then to remaining radicular root [39].

Mechanical properties vary between different types of posts. An ideal post-core foundation should have superior fracture resistance that is higher than mastication forces. While other mechanical properties such as elastic modulus, coefficient of thermal expansion and compressive strength must be same as those of radicular dentin [39]. Previous studies suggested that the most appropriate post space diameter for maxillary central incisors should range from 1.3 mm to 1.7 mm or order to maintain post stability as well as not to weaken the residual radicular dentin [23]. Also, it has been reported that the post diameter is more crucial than its length regarding its fracture resistance; the increase of post diameter can enhance its fracture resistance but unfortunately it reduces the sustaining resistance of remaining tooth structure due to the excessive removal of sound dentinal root [23].

This study investigated the fracture resistance of endodontically treated maxillary central incisors, restored with two post-core systems in 1.3-mm (narrow) and 1.75-mm (wide) post space diameters**.** The null hypothesis of this study, which claimed that there would be no significant difference between fracture resistance of the studied groups, which was accepted in light of this study’s findings.

Thus in this study there was no statistically significant difference among the four groups. Although, mean fracture resistance was in its highest value in group GW (638.7 ± 285.1 N), followed by group ZW (598.5 ± 127.6 N), then GN group (442.8 ± 65.38 N). The lowest mean fracture resistance was recorded in group ZN (435.3 ± 117.3 N).

The highest fracture resistance was found in subgroup GW; glass fiber posts with 1.75 mm diameter, with mean of 638.7 ± 285.1 N. This high value is attributed to that the modulus of elasticity of glass fiber posts is almost same as that of radicular dentin. This supported the presence of a monoblock that aided in dissipation of forces along the long axis of the post itself, thus high resistance of the roots against fracture when subjected to occlusal forces. This came in agreement with a study by Habibzadeh et al. [10]

In accordance with the results of the current study, previous studies also reported that increasing the post diameter enhances its fracture resistance. They reported value of 514.2 ± 136.3 N, which is comparable to the findings of the present study. However, this leads to the excessive removal of sound tooth dentin and hence weakening of the residual tooth structure occurs [23, 40–43].

Kul et al. explained the most probable reason causing the variety in mode of failure. They reported that the low modulus of elasticity of glass-fiber post closely reaches that of dentin. While zirconia posts have high modulus of elasticity. The low modulus of elasticity aided in the absorption of forces along the length of fiber posts as well as its ability to bond to the tooth dentin; providing a monoblock effect, greatly reducing the force transmission to the tooth leading to lower risk of fracture. That also came in agreement with the results of the current study [44].

Previous studies by Saritha et al., Kurthukoti et al. and Shukla et al. compared fracture resistance of zirconia posts and glass fiber posts of endodontically treated teeth. They proved that fracture resistance between tested groups was not significant (*P* > 0.65) and zirconia and fiber-reinforced posts showed high fracture resistance values which came in accordance to the findings of the current study [37, 39, 45].

On the contrary, Habibzadeh at al., evaluated the fracture resistances of zirconia and fiber-composite post systems under all-ceramic crowns in endodontically treated teeth. Fiber-glass posts with composite cores showed the highest fracture resistance values (915.70 ± 323 N), while the zirconia post-system showed the lowest resistance (435.34 ± 220 N). The differences among the groups were statistically significant (*P* < .05) for the zirconia group, as tested by ANOVA test. That might be attributed to the crown restoration, as it may have absorbed the occlusal forces then dissipating them to the core and post, leading to less stress accumulation over the post and core [10].

Özarslan et al. investigated the fracture resistance and fracture mode of maxillary central incisors restored with two different diameters and three different post-core systems. Their study revealed that the highest fracture strength was found in Group F; teeth restored with glass fiber posts, followed by group Z; teeth restored with zirconia custom-made posts. Their findings showed statistically significant differences existed between the fracture strengths of the post materials, which came in contrary to the findings of the current study. That might be explained by the different types of zirconia used as well as the glass-fiber post, in addition to the minute difference in post space diameters in that study; 1.4-mm and 1.6-mm [23].

Subgroup ZW; custom-made zirconia posts with 1.75 mm diameter, showed a slightly lower mean fracture resistance than subgroup GW. This is supported by a previous study by Alkhatri et al. and Abduljabbar et al., as they reported that CAD/CAM zirconia posts had high fracture resistance due to the chemical stability, high elastic modulus, and high toughness in conjugation with mechanical strength [13, 46].

Regarding zirconia post-core unit, Prettau Zirkonzahn was used in this study. Prettau Zirconia is partially stabilized with yttrium and enhanced with aluminum. This aided in increasing mechanical properties; high flexural strength up to 1200 MPa, along with stable shrinking feature allowing the optimum precision [47]. This suggested the reason of the ability of teeth restored with zirconia posts to have high values of fracture resistance but certainly less values than glass-fiber posts subgroups.

All samples restored with zirconia post-core system showed non-favorable mode of fracture. This came in correlation with the variation between elastic modulus of zirconia post, which is high, and the radicular dentin that varies between 12 and 14 MPa [39]. This variation inhibited forces distribution along the long axis of the tooth and dissipation of forces among the roots was not homogenous due to the different rigidity of the components [46]. This came in agreement with a study by Salameh et al. and Lassila et al. who discussed the difference of rigidity between components and its impact on forces’ dissipation. They gave an explanation regarding teeth failure that zirconia posts didn’t dissipate or absorb occlusal forces, but instead it was totally transferred upon tooth structure itself; forces were transmitted from the high rigidity component to the less rigid component [48, 49].

Previous studies evaluated flexural strength (900–1200 MPa) of zirconia posts and high modulus of elasticity of 200 MPa which prevent the plastic manner of the material to be presented; resisting high forces but without dissipation. This led to failure of teeth in a non-restorable manner; vertical root fracture that end by extraction of the restored teeth [10, 50, 51]. This explains that ability of zirconia posts to sustain forces and provide high fracture resistance and failure at high force application; but with catastrophic failures.

Subgroup GN showed lower values of fracture resistance as they had a narrower diameter that couldn’t resist forces and failed at less exerted forces. However, failures were confined to the core foundation only, without any damage to the tooth itself. This came in agreement with a study by Özarslan et al. [23].

A study by Beck at al. reported that fracture resistance of glass-fiber posts was not significantly different to that of custom-made zirconia one piece post and core. They also reported that catastrophic failures most often occur with zirconia posts and restorable fractures occurred with glass-fiber posts. That came in accordance to the findings of this study as all samples of group G showed favorable fractures that were confined to the core material only or barely reaching the post itself, while zirconia posts showed catastrophic failure that unfortunately end by tooth extraction [52].

This study’s findings were also in agreement with a study by Habibzadeh et al., Gu et al. and Akkayan and Gülmez stated that fractures associated with zirconia posts were mostly non-restorable, while these associated with glass-fiber posts were more reparable. They came out with an explanation that the high rigidity as well as the high elastic modulus of zirconia posts transfer subjected forces directly to the tooth without any kind of dissipation or absorption by the post-core unit, and that was the main reason causing the fracture of restored teeth. That also came in agreement with the findings of the current study [10, 53, 54].

Limitations of the present study is the lower sample size and the lack of thermal changes that are present in the oral environment and masticatory forces were not applied as it is an in-vitro study, which does not reflect the oral cavity conditions as well as the absence of cyclic loading of the specimens before testing which might alter the results. So, further in-vivo studies are required to evaluate fracture resistance of different esthetic posts in oral environment for successful prosthodontic procedures. So the use of chewing stimulator, crown restoration and cyclic loading of the samples is recommended to obtain more consistent results to the clinical situation. That’s because in the current study, a universal testing machine with a static manner was used to measure fracture resistance of specimens. Furthermore, the teeth collected for this study were selected according to the preferred post sizes. The different canal configurations, post drills and posts themselves may have affected the homogeneity of the cement thickness between the post and root canal wall. Also, a larger sample size is recommended in further in-vitro studies to obtain a more accurate significant difference among the groups in future studies.

## Conclusion

Based on the findings of this study, we conclude that:

1. There is no statistically significant difference between fracture resistance of endodontically treated teeth restored with either zirconia post-core or glass fiber post and composite resin core in different post space diameters. Highest was presented in group GW and lowest was exhibited in group ZN.
2. The increase in post diameter increases the fracture resistance of the post itself, however, it greatly affects the fracture resistance of remaining radicular dentin.
3. Modulus of elasticity is a major contributing factor that affects the fracture resistance of endodontically treated teeth and their mode of failure that occurs.
4. Teeth restored with glass fiber posts and composite resin core can be retreated after their favorable failure. On the other hand, teeth restored with custom-made zirconia post-core require extraction later on as their failure extends beyond the cervical line and involves vertical fracture along the length of the root.

## Data Availability

The datasets generated and analyzed during the current study are available from the corresponding author on reasonable request.

## Abbreviations

mm: millimeter
mW/cm2: milliwatt per square centimeter
nm: nanometers
ISO: the International Organization for Standardization
°C: Celsius or centigrade
CAD/CAM: computer-aided design & computer-aided manufacturing
no: number
NaoCl: Sodium hypochlorite
ED5x: 5-axis simultaneous dental mill
mm/min: millimeter per minute
IBM SPSS: software for advanced statistical analysis
*P*: probability
N: Newton
MPa: megapascal
